# Secondary Metabolite Localization by Autofluorescence in Living Plant Cells

**DOI:** 10.3390/molecules20035024

**Published:** 2015-03-19

**Authors:** Pascale Talamond, Jean-Luc Verdeil, Geneviève Conéjéro

**Affiliations:** 1 Institut des Sciences de l’Evolution Montpellier ISE-M, Université Montpellier, CNRS, IRD, EPHE, CC 065, Place Eugène Bataillon, 34095 Montpellier, France; 2 Histocytology and Plant Cell Imaging platform PHIV, UMR AGAP (CIRAD, INRA, SupAgro)-UMR B&PMP (INRA, CNRS, SupAgro, Montpellier University), 34095 Montpellier, France; E-Mails: verdeil@cirad.fr (J.-L.V.); genevieve.conejero@supagro.inra.fr (G.C.)

**Keywords:** autofluorescence, secondary metabolites, phenolic compounds, hydroxycinnamic acids, plant tissue, spectral imaging, Linear Unmixing

## Abstract

Autofluorescent molecules are abundant in plant cells and spectral images offer means for analyzing their spectra, yielding information on their accumulation and function. Based on their fluorescence characteristics, an imaging approach using multiphoton microscopy was designed to assess localization of the endogenous fluorophores in living plant cells. This method, which requires no previous treatment, provides an effective experimental tool for discriminating between multiple naturally-occurring fluorophores in living-tissues. Combined with advanced Linear Unmixing, the spectral analysis extends the possibilities and enables the simultaneous detection of fluorescent molecules reliably separating overlapping emission spectra. However, as with any technology, the possibility for artifactual results does exist. This methodological article presents an overview of the applications of tissular and intra-cellular localization of these intrinsic fluorophores in leaves and fruits (here for coffee and vanilla). This method will provide new opportunities for studying cellular environments and the behavior of endogenous fluorophores in the intracellular environment.

## 1. Introduction

When excited by UV/Vis radiation of suitable wavelengths, we can observe the fluorescence emission from endogenous fluorescent molecules in living tissue (algae, plant and animal). This fluorescence emission, called autofluorescence, is an intrinsic property of cells. Nevertheless, one must be able to distinguish these intrinsic signals from other fluorescent signals resulting from the addition of exogenous markers [1]. Although the intensity of autofluorescent markers is quite low, it can interfere with the signals of the exogenous fluorescent markers used in typical spectral imaging experiments. In plant tissues, the extracellular matrix and cellular components often contribute to autofluorescence emission to a greater extent than exogenous fluorescent markers [2]. Fluorescence imaging in green plants is unusually challenging because of the large amounts of intracellular fluorophore molecules [3]. Chlorophyll, the main photosynthetic pigment of plants, absorbs the majority of light and emits a strong fluorescence. Nevertheless, this potentially annoying property can be used as an advantage in spectral imaging for obtaining information on their localization and functions on living plants.

Advanced research in imaging spectral microscopy has focused on increasing the discrimination between distinct fluorophores with highly overlapping emission spectra and thus enhance the possibilities of multicolor imaging [1,4]. After no less than one decade of development, imaging method using spectral analysis coupled with advanced Linear Unmixing has become a vital biological imaging tool that combines the advantages of optical imaging and spectroscopy [1,2]. This label-free imaging capability of microscopy methods can reveal autofluorescence emissions from the endogenous fluorophores in living cells and tissues [5]. We developed an approach in imaging spectral coupled to Linear Unmixing which has to ability to gather of spectral profiles about endogenous flurorophores enabling the detection and differentiation of mixed fluorophores in green plant tissues. 

UV excitation (with wavelengths from 340 to 360 nm) of green leaves generates two types of fluorescence: a blue-green fluorescence and red and far-red fluorescence [3,6,7]. Altogether, the red and far-red fluorescence of plant cells are called “chlorophyll fluorescence” because they correspond to the sole intrinsic fluorophore in plant leaves with emission in this part of spectrum. The blue-green fluorescence has long been identified, but it has not fostered much investigation and it has not been systematically studied until recently. Unlike the red fluorescence, which is almost all derived from chlorophyll, the blue fluorescence of plants combines the emissions of several universal cellular flurophores [8,9] including: nicotinamide (NADH), flavin (FMN, FAD) coenzymes, pyridoxal phosphate (vitamine B_6_), folic acid (vitamin B_9_) and secondary metabolites including the three main groups (phenolics, alkaloids and terpenoids). This fluorescence is characterized by a wide spectral emission band in the blue band (430–450 nm) and a shoulder in the green band (520–530 nm) [6]. From an intact leaf, the detected fluorescence is emitted mainly by compounds located in the walls of epidermal cells and vascular bundles [10,11,12]. 

**Figure 1:**
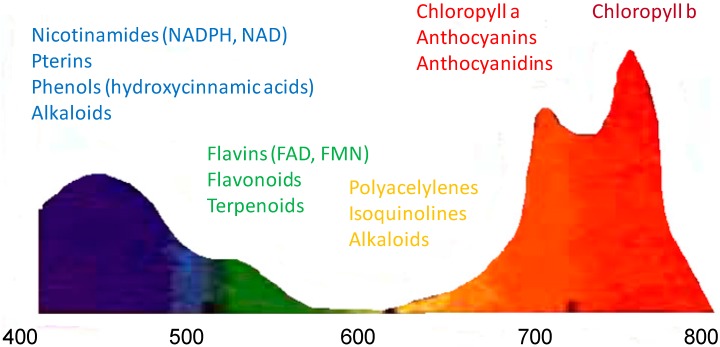
Fluorescence emission spectrum of a typical green leaf under UV-radiation (λ_exc._ = 355 nm). The fluorescence bands in blue (430–450 nm), green (520–530 nm), red (680), and far-red (740 nm) regions, adapted from [6].

Secondary metabolites refer to a generic term used for more than 200,000 different substances that are exclusively found in plant and sometimes in fungi [13]. Plants produce a vast array of secondary metabolites in response to interactions with their environment, which impart flavor, color, bitterness, astringency and fragrance, and confer protection through a variety of antimicrobial, pesticidal and pharmacological properties. Some flavonoids and coumarins (chromones), pterines (pyridoxal coenzyme), some phenols (hydroxycinnamoyl acids) and some alkaloids (caffeine) in plant emit in the blue region (450–500 nm), flavins and some terpenoids in the green region (500–530 nm), while polyacetylene, isoquinoline and some alkaloids emit in the yellow and orange regions of the visible spectrum and anthocyanins and anthocyanidins emit in the red region of the visible spectrum [14,15,16,17] (Figure 1). 

Metabolites and enzymes involved in the biosynthesis of phenylpropanoids, terpenoids and alkaloids have been localized in a variety of plant cells. Methods for detecting the tissue-specific distribution of phenolic compounds have been developed by coupling microspectrofluorometry and multispectral fluorescence microimaging or by confocal laser scanning microscopy [18,19,20]. These methods enabled the discrimination of the relative abundance of hydroxycinnamic derivates and flavonoids in different layers of plant leaves. However, the cell type-specific localization of most secondary metabolic pathways has not yet been characterized. Several physiological functions have been hypothesized, but little is known about the biosynthesis and metabolism of these bioactive compounds in plants. 

Here, the performance of these methods was demonstrated for plants, focusing on leaf and fruit tissues, *i.e.*, the adaxial and abaxial epidermis, and palisade and spongy mesophyll. Developments in microscopic techniques, such as confocal laser scanning microscopy, provide a unique opportunity for studying tissue localization of phenolic compounds more precisely than with conventional fluorescence microscopy [21]. Furthermore, multiphoton microscopy outperforms confocal microscopy, due to its deeper tissue penetration and reduced phototoxicity [22]. Here, we describe how this method allowed a finer discrimination of the relative abundances of secondary metabolites in particular, phenolic compounds in different layers of tissues, namely: (1) chlorogenic acid (5-caffeoyl-quinic acid, 5-CQA)), mangiferin (C2-β-d-glucoside-1,3,6,7-tetrahydroxyxanthen-9-one) and caffeine (1,3,7-trimethylxanthine) in coffee leaves and (2) vanillin glucoside (4-*O*-(3-methoxybenzldehyde)-β-d-glucoside) in vanilla fruit.

## 2. Results and Discussion

### 2.1. Autofluorescence of Living Plant Cells and Tissues

**Figure 2:**
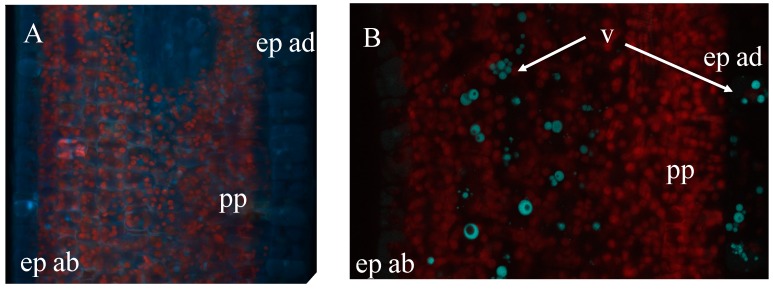
Autofluorescence of fresh young leaf in a cross-section of *Coffea canephora* observed with a epifluorescence microscope (**A**, filter exc: 340–380 nm, em: 425–800 nm) and with a multiphoton microscope (**B**, exc: laser IR 720 nm, em: channel 435–485 nm in blue, channel 670–700 nm in red) ep: epidermis, pp: palisade parenchyma, v: vesicle.

Endogenous fluorophores are particularly abundant in plant tissues and are involved in structural and metabolic functions at cell and tissues level [8,9,23]. Several studies have been performed, both *in vivo* and *in vitro*, on the use of autofluorescence-based techniques, in particular studies with fluorescence microscopy, for the discrimination the tissues. The basic fluorescence microscope irradiates the tissues with a desired and specific band of wavelength and then to separate the much weaker emitted fluorescence (Figure 2).

A cross-section of a live young leaf of *Coffea canephora* observed with an epifluorescence miscroscope was found to emit two types of autofluorescence under UV light at 340–380 nm: (1) a red fluorescence of chlorophyll in chloroplast, originating from the palisade parenchyma cells; and (2) a white-blue fluorescence originating from some vesicles and vacuoles in the mesophyll cells and the epidermis (Figure 2A). The same sample observed with a multiphoton microscope at 720 nm excitation (UV-like excitation light) gave a better resolution to identify the subcellular anatomy (Figure 2B) and avoided the phototoxicity of the UV lamp usually used with a wide-field microscope. The relative intensities of this blue fluorescence are highly sensitive and species-specific and they further depend on environmental factors [8,14]. The fluorescence emission spectrum induced by UV radiation in a particular plant can be considered as a molecular signature that reflects environmental (biotic or abiotic) effects and can reveal the significance of secondary metabolites. In this region of the UV spectrum, the intracellular fluorophores that are most often encountered are universal in plants such as pyridinic and pterine coenzymes, pyrodoxal-5'-phosphate (PLP) and some phenolic compounds and alkaloids. Hydroxycinnamic acid esters (HQA) and xanthine derivates are the principal secondary metabolites found in the leaves of commercial coffee trees. In young leaves, the HQA esters represent 0.3% to 7.8% of the dry weight depending on plant species [24,25]. In *C. Canephora* leaves, hydroxycinnamic acid esters are the main secondary metabolites and they could be responsible for the absorption maximum around 450 nm [8,26,27], as other fluorophores absorbing at other frequencies are less abundant. In order to interpret this pattern; it is crucial to understand the respective contributions of every fluorophore to the autofluorescence signature. 

### 2.2. Spectral Signatures of Endogenous Fluorophores

The relatively high-transmission spectral region between ~400–800 nm is often described as the “optical window” where biological tissues both absorb and scatter light quite strongly. Cells contain molecules that become fluorescent when excited by visual radiation of suitable wavelength. This autofluorescence emission, by endogenous fluorophores is an intrinsic property; therefore the spectrum provides an accurate fingerprint of these molecules. In two-photon microscopy-pulsed (~100 femtoseconds) infra-red laser light is used to excite fluorophores that can absorb visible light [28,29] Excitation is restricted to a very tiny volume in the sample (one femtoliter). The laser intensity in the focal volume is so high- that each fluorophore absorbs two photons, in contrast to conventional fluorescence where a single photon is absorbed. With two photon excitation, only the focal volume is excited and the out-of-focus volume is unaffected [30]. Laser light, in the range 700 to 1050 nm, is scattered to a much lesser extent than visible and UV wavelengths, it penetrates at least twice deeper into plant tissues (~500 μm) and thus allows for 3D imaging of thicker specimens [28]. In addition, the combined energies of two low-energy photons are equivalent to the energy of one high-energy photon. Single-photon spectra (absorption, emission) are well documented for a wide range of molecules [31]. However, the increasing use of non-linear miscroscopy, which relies on multiphoton technology, requires knowledge of multiphoton photophysics and especially the two-photon excitation properties of fluorophores and biological molecules. It can be presumed that these spectra do not differ from those of single-photon spectra, but experimental evidences are lacking. Therefore, experiments were conducted to ascertain that the spectral characteristics of single and two-photon excited autofluorescence were identical. 

**Figure 3:**
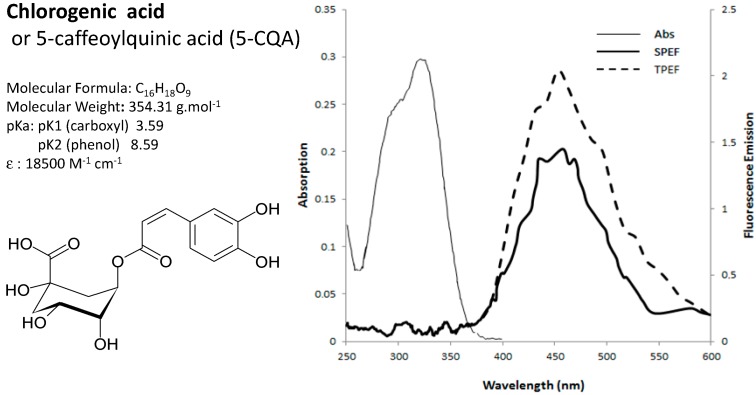
Absorption and emission spectra of chlorogenic acid (5-caffeoylquinic acid, 5-CQA) with conventional spectrometer (single photon excited fluorescence, SPEF) and multiphoton laser (two-photon excited fluorescence, TPEF) and the chemical characteristics.

Conventional spectroscopy was achieved using pulsed Xenon light source. The two-photon emission spectral signatures were obtained by spectral acquisition λ_exc_ = 720 nm. The detection bandwidth was set to collect emissions from 365 to 700 nm, using a specific PMT (photomultiplier tube) detector. The spectral characteristics of single-photon excited fluorescence (SPEF) and two-photon excited fluorescence (TPEF) signals were obtained for chlorophyll(a), chlorogenic acid, caffeine, mangiferin and vanillin 4-*O*-β-d-glucoside, which are the major fluorophores in coffee leaves (*C. canephora*, *C. pseudozanguebariae*) and vanilla fruits (*Vanilla planifolia* Jackson ex Andrews), respectively, and are shown in Table 1. The absorption spectra with two-photons are much broader than those with a single-photon. This could explain the red shift in emission maximum.The excitation wavelengths were selected as a function of the available laser equipment and were as close as possible to the optimal excitation wavelengths of the fluorophores under study. With TPEF at 720 nm, the excitation wavelength is too distant from optimal and the fluorescence intensity of caffeine is at least 100 times weaker than at the optimal wavelength of 272 nm (SPEF). The full absorption and emission spectra of chlorogenic acid were acquired in the spectral range between 250 and 600 nm (Figure 3). 

**Table 1:** Spectral regions of absorption and emission maxima with conventional spectrometry and multiphoton laser for five endogenous fluorophores.

Metabolite	Conventional Spectrometer		Multiphoton Laser
	λ_absorption_	λ_emission_	λ_emission_
	Maxima (nm)	Maxima/Bandwidth (nm) *	Maxima/Bandwidth (nm) *
Chlorophyll a	430	685/22	660/35
Chlorogenic acid	324	455/90	453/105
Methoxybenzaldehyde	318	424/90	515/115
Mangiferin	318, 366	nd	570/80
Caffeine	272	397/80	405/60

* λ_max_/bandwidth of fluorescence emission that is measured by the width of spectral profile at 50 percent of the maximum yield.

No differences between SPEF and TPEF emission spectra were found for the peak λ_max_ and bandwidth of four endogenous fluorophores: chlorophyll, chlorogenic acid, mangiferin and caffeine. By contrast, the SPEF and TPEF signals of vanillin 4-*O*-β-d-glucoside excited at 300 nm and 720 nm exhibited about 90 nm redshift and bandwidth broader compared with the SPEF spectra. In general SPEF and TPEF spectra are identical (e.g., [32]) but some studies found a redshift between the SPEF and TPEF spectra, for example for 7-hydroxycoumarine [33], another phenolic compound, and for pure coenzyme fluorophore (NADH) [34]. The reasons behind these spectral differences remain to be elucidated. 

### 2.3. Phenolic Compounds Localization in Coffee Leaf

**Figure 4:**
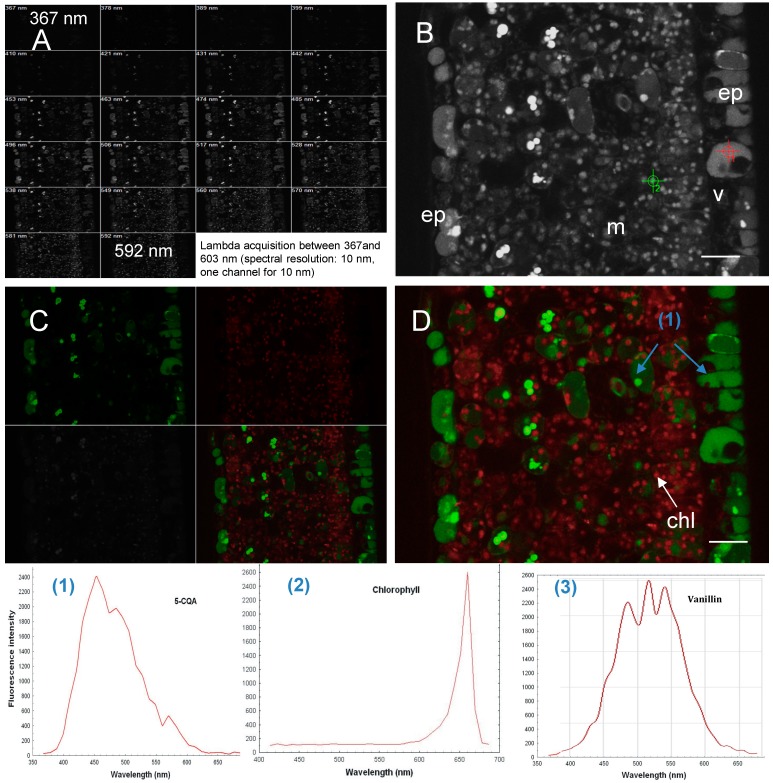

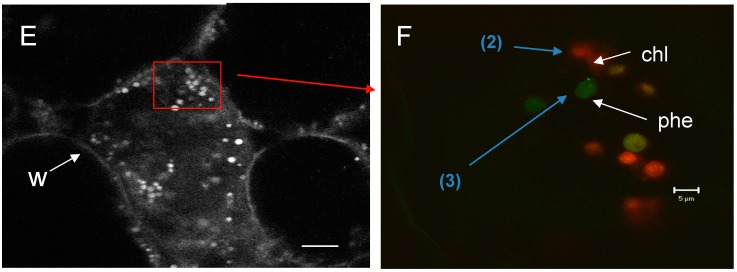
Spectral imaging and analysis in cross sections of *Coffea canephora* young leaf and *Vanilla planifolia* fruit by multiphotonic microscopy, lambda excitation: 720 nm. (**A**) Images gallery obtained after lambda acquisition between 367 and 603 nm (spectral resolution: 10 nm, one channel for 10 nm); (**B**) Spectral acquisition, overlay of different channels of A, named Lambda Max; (**C**) Linear Unmixing, (left: split, right: overlay of 3 channels); (**D**) Linear Unmixing with emission spectra of chorophyll (red) and 5CQA or chlorogenic acid (green) in *Coffea canephora* young leaf: overlay image of three channels and corresponding to fourth image of C; (**E**) Spectral acquisition (**F**) Linear Unmixing with emission spectra of 4-*O*-(3- methoxybenzaldehyde)-β-d-glucoside (green) and chlorophyll (red) in inner placenta cells of *Vanilla planifolia* pods. The spectral images are false color. Reference emission spectra of 5CQA (**1**), chlorophyll (**2**) and vanillin 4-*O*-β-d-glucoside (**3**). (chl, chloroplast; ep, epidermis; m, mesophyll; phe, phenyloplast; w, cell wall; v, vacuol). Scale bar: A and B: 20 µm; C: 50 µm; D: 5 µm.

Using a multiphoton microscope set at a 720 nm excitation wavelenght, Linear Unmixing generated the image illustrated in Figure 4. This method uses an array of photomultiplier tubes and a polychromatic multichannel detector between 360 and 700 nm. The Linear Unmixing method compares the measured spectrum, which can be composed of multiple fluorescent components to a library of reference spectra obtained from the analysis of pure compounds. We fitted the measured emission signal at each image pixel with the function I(λ) sum, computed according to the equation in given in Section 3. A composite image was generated from four base images, two of which corresponding to the two pure (reference) compounds, and the third one to the residual fluorescence channel (Figure 4C,D). Linear Unmixing separates the summed spectral data into individual images for each reference fluorophore, by using the least square difference principle applied at the pixel level [35].

The spectral signature is determined for each pixel of the scanned image and is used for the subsequent digital separation of fluorescent molecules. Then, the Linear Unmixing process enables the discrimination of between various fluorescence signals, even between those with widely overlapping emission spectra. Reference spectral signatures were acquired on the microscope using controls of standard powders known to be present in a *C. canephora* and *C*. *pseudozanguebariae* leaf blade cross-section. Each control molecule (in the case of coffee leaves, chlorogenic acid, caffeine, mangiferin and chlorophyll spectrum) is carried out with the same excitation wavelength (720 nm) and exactly the same optical conditions. The spectral signature was specific for every molecule and stored in the Spectra Database. 

Since reference λ-stacks are determined by the spectral properties of the respective fluorophores and are independent of their concentrations, they can be used to assemble a matrix fluorophore with specific weighting factors, which is subsequently used to determine the respective contributions of each fluorophore to each image pixel [1]. The sensitivity of the method depends directly on the sensitivity of the detector and spectral characteristics of the fluorophores under study. The spatial resolution is determined by pixel size (here ~0.4 µm^2^) under our experimental conditions. Caffeine is not detected in epidermal cells because its fluorescence intensity is too weak for this particular excitation wavelength and too far away from its nominal wavelength. Chlorogenic acid (5-CQA) and mangiferin both exhibited a broad spectral range with peaks at about 425 and 520 nm, respectively [27]. Fluorophores occur mainly in epidermis cells and particularly in vesicles (Figure 4B). 

Mangiferin fluorescence appeared in small vesicles of the parenchyma and in vacuoles of the abaxial epidermis, but not in the adaxial epidermis [27]. By contrast, 5-CQA fluorescence was abundant in the abaxial epidermis and constituted an important reservoir of phenolic compounds, but its role has not been clearly defined yet. In sun leaves of *Phillyrea latifolia*, flavonoids largely accumulated in the adaxial epidermal layer and subepidermal cells [19]. The localization of these phenolic compounds in the vacuoles of epidermal cells, and in the vesicles and chloroplasts of mesophylic cells further supports the hypothesis of their involvement in leaf protection against UV damages or pathogens [20,26,27].

To date, phenolic compound histolocalization methods mainly uses the standard reagent for phenols and flavonoids, the Neu’s reagent, and then examination by epifluorescence microscopy [36,37]. Excitation with UV laser beam at 364 nm induced strong green fluorescence in the epidermal cells of coffee leaves, which was attributed to the presence of phenolic compounds [24]. In juvenile leaf blades, an intense greenish-white fluorescence was observed in all chlorenchyma cells, including the palisade and spongy mesophyll cells [24]. Under UV-light, in absence of any interference with the intrinsic red fluorescence of chlorophyll, detection of phenolic compounds and derivatives from hydroxycinnamic acid esters, in particular ferrulic and caffeic acids, was possible. Another phenolic compound, the mangiferin (*C-*glucosylxanthone), a yellow-pigment found in wild coffee species does not require any reagent and was directly observed though autofluorescence [38,39]. The young leaves of wild coffee species (*Coffea pseudozanguebariae*) showed a yellow and orange fluorescence under UV light [39]. In a typical spectral imaging, the potential of spectral microscopy has been broadly demonstrated by the use of emission spectral signature in living plant cells marked different fluorophores. There are usually several fluorophores present in sample not to determine the relative contribution from each endogenous fluorophores. Our approach is different and novel because our method detects only on a base of this specific spectral analysis for discriminating between multiple endogenous fluorophores.

### 2.4. Phenolic Compound in Vanilla Fruit

As was the case for coffee, the histolocalisation of phenolic compounds in vanilla fruit with a Schiff’s reagent produced a fuschia stain, indicating that they were filled with an aldehyde-bearing substance. This response enables no accurate distinction between various aldehydes and is not informative as whether theses aldehydes differ from glycosylated 4-*O*-(3-methoxybenzldehyde) form. The autofluorescence was characterized to localize and 4-*O*-(3-methoxybenzaldehyde)-β-d-glucoside and chlorophyll in vanilla fruit. The aforementioned procedure was applied to inner mesocarpal cells using two reference spectra, *i.e.*, the responses of chlorophyll and 4-*O*-(3-methoxybenzldehyde)-β-d-glucoside excited at 740 nm [40]. Linear Unmixing was applied by using the reference spectra and deducing the flurophores at the pixel’s level. The result of the Linear Unmixing showed that the content of some like-chloroplast organelles in vanilla fruit was 4-*O*-(3-methoxybenzldehyde)-β-d-glucoside, while some others contained both only chlorophyll, or both chlorophyll and this glucoside (Figure 4E,F). Thus, it appears that the storage of 4-*O*-(3-methoxybenzldehyde)-β-d-glucoside varies between chloroplasts in the inner mesocarp of vanilla fruit. Spectral analysis combined with biochemical data and immunocytological and electron microscopy studies allowed us to identify a new chloroplast-derived organelle, the phenyloplast, that stores an important phenolic compound [40].

Spectral analysis provides a number of important advantages: it allows a molecule to be tracked in the living material by limiting artifacts related to sample preparation. Unlike the situation with histochemical staining reagents, it can locate fluorophore by its specific spectral signature. Spectral imaging and Linear Unmixing extends the possibilities of obtaining specific strong and sustainable signals without degradation of cellular functions in living tissue.

## 3. Experimental Section

### 3.1. General Experimental Procedures

Epi-fluorescence microscopy was carried out on a Leica DM 6000 instrument equipped with a Q-Imaging camera (long-pass filter 425 nm, QImaging, Surrey, BC, Canada). A Zeiss 510 META NLO multiphoton microscope (Zeiss, Jena, Germany) equipped with a Coherent Chameleon Ultra II laser was used to obtain emission fluorescence from fresh leaves. A 25×/0.8 Plan-Neofluar immersion objective was used to obtain galleries of spectral images and emission spectra from fresh plant sections. The multiphoton microscope with tunable laser Chameleon Ultra II (690–1080 nm range excitation, Coherent, Santa Clara, CA, USA) enables the excitation of secondary metabolites in a manner similar to an UV laser. Optimal excitation was obtained at λ = 720 nm and band-pass emission in the 365–700 nm range using an array of 32 photomultiplier tube (PMT) detectors (Zeiss), each with a 10.7 nm bandwidth. This procedure was performed on live tissue cross-sections, as well as on pure standards for: chlorogenic acid (5-CQA, 5 caffeoyl-quinic acid), caffeine (1,3,7,-trimethylxanthine) (Sigma-Aldrich, St Quentin Fallavier, France), mangiferin (C2-β-d-glucoside-1,3,6,7-tetrahydroxyxanthen-9-one) (Extrasynthese, Genay, France), and 4-*O*-(3-methoxybenzaldehyde) β-d-glucoside (Sigma-Aldrich) powders. The chlorogenic acid (5-CQA) in a methanol solution in was measured on a FL-2500 fluorescence spectrophotometer (Hitachi, Tokyo, Japan). The excitation wavelength was 320 nm and the emission wavelength was within the 250–600 nm range. 

Spectral analysis was carried out using the advanced Linear Unmixing function (LSM 510 software, Zeiss) which that separates mixed signals pixel by pixel using the entire emission spectrum of each defined autofluorescent compound in the sample. This function requires at least two spectra and was applied with the advanced iterative option and one residual channel. After spectral imaging acquisitions on leaf cross-sections, the advanced Linear Unmixing function allowed visualization with coded colours of the fluorescence of each standard and chlorophyll in cell based on their reference spectra.

### 3.2. Processing Spectral Images

The fundamental concept underlying Linear Unmixing calculations is each pixel in the spectral image is categorized as representing a mixture of fluorophore signals (intensities) when the measured spectrum (I(λ)) can be deconvolved into the proportion, weight, or concentration (C) of each individual fluorophore reference spectrum (R(λ)) when the values are summed. Thus, each reference spectrum of a pure fluorophore is described as Ri(λ) where *i* = 1,2,3.....n represents the index of the fluorophore (Ci). For a particular number of fluorophores (n), this relationship can be represented as [35]:

I(λ) = C1xR1(λ) + C2xR2(λ) + C3 × R3(λ) +........ + Cn × Rn(λ) Or more simply: I(λ) = ∑i Ci × Ri(λ)

In practice, the signal intensity for each pixel (I) in the spectral image is determined and recorded during acquisition of the lambda stack and the reference spectra for the known fluorophores are measured independently in separate control specimens labelled with only a single fluorophore, using identical sample preparation techniques and instrument settings. The overall spectral contributions from the various fluorophores in the specimen can then be determined as a simple linear algebra matrix exercise by calculating their individual contributions to each point in the measured spectrum, as described in the equations above. For many of Linear Unmixing software packages, the solution is obtained by inputting reference spectral profiles and using an inverse least squares fitting approach that minimizes the square difference between the measured and the calculated spectra.

### 3.3. Plant Material

In these experiments we used *Coffea* plants from the collection cultivated at the IRD Research Centre, in Montpellier, France. Leaves of *C. Canephora* (DB56, DB57) and *C. pseudozanguebariae* (H65, H70) of various genotypes were taken from trees maintained under tropical conditions (natural daylight, 25 °C, 28 °C, 78%–82% humidity). Vanilla (*Vanilla planifolia* Jackson ex Andrews) from the Réunion Island, France, was hand-pollinated. Sound fruits were harvested 4 months and 7 months after pollination and air-freighted in a refrigerated box and delivered to laboratory within 2 days after hand-picking. 

### 3.4. Leaf and Fruit Cross-Sections

Leaf and fruit cross-sections (50 µm) were obtained using HM 650 V vibrating blade microtome (Microm, Waldorf, Germany). Cross-sections were dipped in 0.1 M phosphate buffer salin with ascorbic acid to prevent oxidation.

## 4. Conclusions

Visualization of secondary metabolites of plant by spectral analysis of their autofluorescence under UV-like laser is a novel approach to their localization [26,27,40,41]. The autofluorescence of endogenous fluorophores is used to enable their localization in plant cells and tissues. This was achieved with a special detection system, using spectral analysis, coupled to advanced Linear Unmixing method. This method enables the discrimination of various fluorescence signals, even with widely overlapping emission spectra. The ability to carry out measurements on live tissues without any preparation treatment, such as fixation or staining makes autofluorescence-based techniques of utmost interest for obtaining information on cell morphology, behavior, and metabolism [42]. This approach requires a good knowledge of anatomy of sample and must be correlated with biochemical data about studied molecules. Thereby, the potential for this approach to improve our understanding of bimolecular interaction dynamics is huge and allowing to explore new biology questions not only in plants. 
